# Online public concern about allergic rhinitis and its association with COVID-19 and air quality in China: an informative epidemiological study using Baidu index

**DOI:** 10.1186/s12889-024-17893-4

**Published:** 2024-02-02

**Authors:** Yi Yu, Qinzhun Zhang, Xinmeng Yao, Jinghua Wu, Jialu He, Yinan He, Huaqiang Jiang, Dongxin Lu, Chengyin Ye

**Affiliations:** 1 Department of Epidemiology and Biostatistics, School of Public Health, Hangzhou Normal University, Hangzhou, Zhejiang 311121 China; 2 Department of Health Management, School of Public Health, Hangzhou Normal University, Hangzhou, Zhejiang 311121 China; 3 Health Management System Engineering Center, School of Public Health, Hangzhou Normal University, Hangzhou, Zhejiang 311121 China

**Keywords:** Allergic rhinitis, Baidu index, COVID-19, Air quality index, Mask wearing

## Abstract

**Background:**

Allergic rhinitis is a common health concern that affects quality of life. This study aims to examine the online search trends of allergic rhinitis in China before and after the COVID-19 epidemic and to explore the association between the daily air quality and online search volumes of allergic rhinitis in Beijing.

**Methods:**

We extracted the online search data of allergic rhinitis-related keywords from the Baidu index database from January 23, 2017 to June 23, 2022. We analyzed and compared the temporal distribution of online search behaviors across different themes of allergic rhinitis before and after the COVID-19 pandemic in mainland China, using the Baidu search index (BSI). We also obtained the air quality index (AQI) data in Beijing and assessed its correlation with daily BSIs of allergic rhinitis.

**Results:**

The online search for allergic rhinitis in China showed significant seasonal variations, with two peaks each year in spring from March to May and autumn from August and October. The BSI of total allergic rhinitis-related searches increased gradually from 2017 to 2019, reaching a peak in April 2019, and declined after the COVID-19 pandemic, especially in the first half of 2020. The BSI for all allergic rhinitis themes was significantly lower after the COVID-19 pandemic than before (all *p* values < 0.05). The results also revealed that, in Beijing, there was a significant negative association between daily BSI and AQI for each allergic rhinitis theme during the original variant strain epidemic period and a significant positive correlation during the Omicron variant period.

**Conclusion:**

Both air quality and the interventions used for COVID-19 pandemic, including national and local quarantines and mask wearing behaviors, may have affected the incidence and public concern about allergic rhinitis in China. The online search trends can serve as a valuable tool for tracking real-time public concerns about allergic rhinitis. By complementing traditional disease monitoring systems of health departments, these search trends can also offer insights into the patterns of disease outbreaks. Additionally, they can provide references and suggestions regarding the public’s knowledge demands related to allergic rhinitis, which can further be instrumental in developing targeted strategies to enhance population-based disease education on allergic diseases.

**Supplementary Information:**

The online version contains supplementary material available at 10.1186/s12889-024-17893-4.

## Background

Allergic rhinitis (AR) is an allergic disease in the upper respiratory system, which has become a global health concern with a high prevalence worldwide, affecting approximately 10%-40% of the world’s population [1, 2]. According to epidemiological studies, nearly 200 million people in China are affected by allergic rhinitis [3, 4]. Along with the growing incidence and prevalence, public’s concern of allergic rhinitis is also increasing in recent years. Traditionally, people had limited access to medical information about allergic rhinitis, mainly through health education from medical professionals. Nowadays, Internet searches enable people to have timely access to a wide range of knowledge about the disease [5–7]. Therefore, active internet searches have not only broadened people’s knowledge about allergic rhinitis, but also helped healthcare professionals monitor and understand people’s interest or behavior related to this disease.

With the increasing use of the Internet, researchers are beginning to use search engine based big data to analyze the public’s search behavior on specific subjects. Notably, big data derived from the Google platform has successfully illuminated trends in the prevalence of allergic rhinitis across different countries [8]. Baidu Search, being the preferred search engine in China, using Baidu to search for medical information has become a well-accepted habit among the majority of Chinese people [9]. Therefore, the Baidu Index emerges as a potent tool for tracking disease and health information-seeking behavior [10]. Furthermore, the Baidu Index finds extensive utility in terms of monitoring and predicting the prevalence of diseases, facilitating targeted and rapid responses from health departments and the mass media. Therefore, Baidu Index can potentially play an important role in healthcare. It complements and extends existing clinical and epidemiological data [11]. The query frequency of keywords associated with specific diseases could be highly correlated with the symptoms presented by patients or their treatment needs. This correlation provides insights into the real disease characteristics of diseases and offers a snapshot of the instant medical demand trends within the population [10, 12–14].

The COVID-19 (coronavirus disease 2019) is an acute respiratory infection disease. The global pandemic of COVID-19 has raised public concern about respiratory health issues. It was worth noting that after the COVID-19 outbreak, to prevent the spread of the virus and its infection among the public efficiently, different countries implemented various physical interventions such as wearing masks and quarantine policies, which were among the effective interventions to reduce the spread of respiratory diseases [15, 16]. Primary prevention not only counteracts the spread of COVID-19, but also blocks allergen exposure [17], and thus substantially affect both the onset and the treatment of allergic diseases.

Evidences have shown that exposure to air pollutants and common allergen can significantly exacerbate allergic rhinitis symptoms or reduce human immune function [18–20]. Allergic rhinitis is generally considered to be associated with air pollution. A series of studies found that allergenic grains or paucimicronic particles carrying allergens can interact with air pollutants such as O_3_, NO_2_, and sulfur dioxide (SO_2_) in atmosphere to irritate the respiratory mucosa of atopic populations, potentially increasing the allergic immune response to allergic diseases [21, 22]. At the beginning of the COVID-19 outbreak, social restrictions in China aimed at curbing the spread of the virus, including the maintenance of a certain social distance, a significant reduction in the movement of people, and the closure of businesses, also had a straightforward impact on reducing the outdoor air pollution [23, 24]. Therefore, taken together, we speculated whether such changes related to social restrictions after the COVID-19 pandemic could have significant and instant impact on people’s Internet searching behavior related to allergic rhinitis.

In this study, we investigated the changes in public attention about allergic rhinitis before and after the COVID-19 pandemic, and explored in detail the correlations between air quality and online searches of allergic rhinitis and wearing a mask, during different stages of the COVID-19 pandemic. We assert that monitoring real-time shifts in public concerns and requirements regarding allergic rhinitis through online search trends can enhance and augment traditional allergic disease surveillance systems. This approach not only supplements existing methodologies but also aids governmental organizations and healthcare providers in effectively disseminating health information and conducting health education aligned with public demands. By leveraging insights from online search behaviors, it becomes possible to facilitate a more dynamic and adaptive approach to public health initiatives.

## Method

### Keyword selection and data

The identification of keywords in this study can be shown in the flowchart in Figure S1 (see Additional file 1). Keywords specific retrieval process is detailed in Additional file 1. A total of 33 keywords were generated for the subsequent analysis. According to allergic rhinitis guidelines and medical practitioners' recommendations [1]. The 33 search terms were further grouped into the four categories of “disease”, “etiology”, “symptoms/complications”, and “disease treatment/management”, which are listed in Table S1 (see Additional file 1). The daily BSIs of each term were extracted and used for subsequent analysis.

The Chinese government announced the closure of Wuhan on January 23, 2020, so we chose this time point to separate the two-year periods before and after the COVID-19 pandemic [25]. Following that, we defined the period from January 23, 2017 to January 22, 2020 as the “pre-COVID-19” segment, while the period from January 23, 2020 to June 23, 2022 as the “post-COVID-19” segment. According to the time when the WHO named the major prevalent variants and they spread in China, “post-COVID-19” has been divided into four periods, named the original-variant (from January 2020 to November 2020), Alpha (from December 2020 to April 2021), Delta (from May 2021 to November 2021) and Omicron (from December 2021 to June 2022) period respectively [26–28]. Original-variant referred to all mutant variants before Alpha. Additionally, in order to understand the relationship between air quality and the public concern about allergic rhinitis or mask-wearing, we choose Beijing as a proxy city, the detailed description is shown in Additional file 2. We also retrieved daily BSIs of 33 allergic rhinitis-related keywords from January 23, 2019 to June 23, 2022 and the “mask wearing” term for the period from January 23, 2020 to June 23, 2022 for consolidation and analysis. The air quality index (AQI) data were obtained from the website of the China Air Quality Online Monitoring and Analysis Platform [29]. From this platform, the daily AQI of Beijing from 23 January 2019 to 23 June 2022 was obtained. AQI is a composite index reflecting the level of air pollution. The daily AQI values usually indicates real-time air pollution and warns people of potentially poor air quality. The higher the AQI value is, the more severe the air pollution is.

### Statistical analysis

To analyze search trends for allergic rhinitis, we summarized the monthly BSI (sum of daily BSIs from the first day of a month to the last day of a month) for all search terms related to allergic rhinitis from January 23, 2017 to June 23, 2022, and plotted curves of search volume for all keywords to describe changes in public attention. Meanwhile, the time series data were seasonally decomposed using the ‘decompose’ function of R software (version 4.1.2) into trend factors, seasonal components and random variables, representing both irregular and periodical changes over time.

To take into account the impact of the number of internet users during a certain period, we extracted the number of Internet users (million) in 2019 and 2020 from the China Statistical Yearbook respectively, and calculated the adjusted daily BSI values by the formula of daily BSI/number of Internet users (million) [30, 31]. The Mann–Whitney U test was implemented to test the null hypothesis of this study, that is, there was no statistically significant difference in the adjusted allergic rhinitis BSIs before and after COVID-19. The median adjusted BSI was used to describe the search volume for each theme of allergic rhinitis. Spearman’s “Rho” correlation was used to measure the association between the values of daily AQI and the daily BSIs related to allergic rhinitis. Statistical analysis was performed using IBM SPSS Version 23.0 (IBM Corporation), and *p*-values < 0.05 were considered to be statistically significant.

## Results

### Temporal analysis of allergic rhinitis BSI in China

**Fig. 1:**
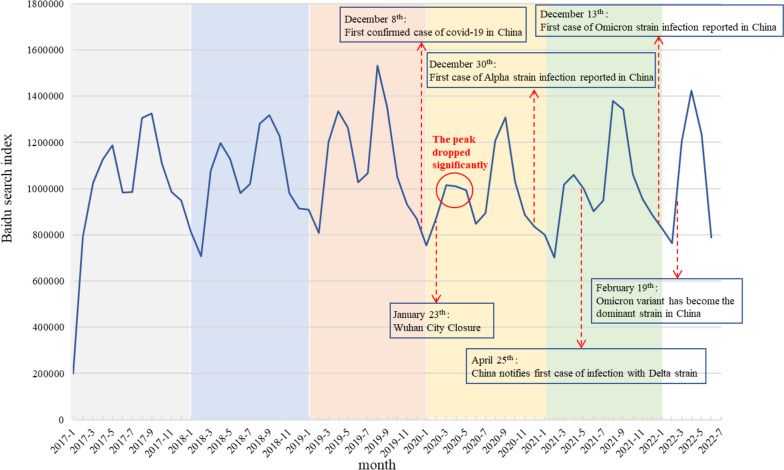
The temporal trend of the total allergic rhinitis BSI from January 2017 to June 2022

We created monthly time-series curves for the total BSI of all allergic rhinitis-related keywords from January 23, 2017 to June 23, 2022 (Fig. 1). Overall, we found there is a discernible seasonal fluctuation in search interest of allergic rhinitis-related keywords. This seasonal fluctuation could be described as two separate peaks from March to May and from August to October. Following the COVID-19 outbreak, the search interest for allergic rhinitis dropped significantly during the first peak period from March to May 2020 compared to the same period in 2017, 2018 and 2019.

**Fig. 2:**
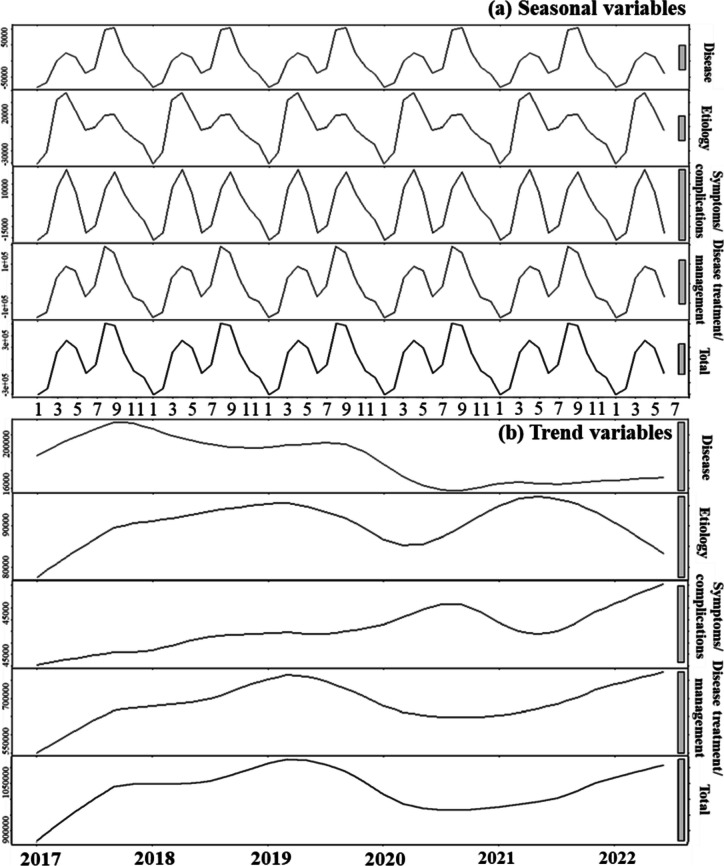
Seasonal (**a**) and trend (**b**) patterns of BSIs for allergic rhinitis by theme and total volumes

After the decomposition, the trend component of the total BSI of allergic rhinitis began to rise rapidly since January 2017, and reached its peak in April 2019. After that, it showed a downward trend in the following months, and hit the bottom in May 2020. Meanwhile, a regular seasonal component with two peaks from March to April and in August was also observed, indicating the seasonality of the public’s search interest for allergic rhinitis (Fig. 2a). We further separately investigated the four themes of allergic rhinitis: “disease”, “etiology”, “symptoms/complications” and “disease treatment/management” (Fig. 2b), and found that the “disease treatment/management” theme revealed similar pattern to the overall searching trends. More details could be found in Additional file 3.

### Public concern about allergic rhinitis Pre-and Post-COVID-19

Comparison of changes in overall search interest for allergic rhinitis between the pre-COVID-19 and post-COVID-19 periods found that the adjusted daily BSI showed a significant decrease in search volume after the pandemic of COVID-19 (25.65 vs 23.48; *p* < 0.001), as shown in Table 1. Among the four allergic rhinitis-related themes, the search interest of the “symptoms/complications” theme increased significantly (1.34 vs 1.66; *p* < 0.001) after COVID-19 whereas the research interest of the “disease” (5.16 vs 3.76; *p* < 0.001), “etiology” (2.27 vs 2.20; *p* = 0.022), and “disease treatment/management” (16.96 vs 15.85; *p* < 0.001) themes all decreased significantly after COVID-19. In detail, the adjusted BSI for the term “symptoms of allergic rhinitis” in the “symptoms/complications” theme has the most significant increase after the occurrence of COVID-19 (*p* < 0.001) (see Additional file 4, Table S2). There are also representative changes in some other keywords as detailed in Additional file 4.

**Table 1:** Comparison of adjusted daily BSI (per million internet users) for different themes of allergic rhinitis before and after the COVID-19 pandemic

Variate	Pre-COVID-19 (2017.01.23–2020.01.22)	Post-COVID-19 (2020.01.23–2022.06.23)	*p*-value
	**M(IQR)**	**M(IQR)**	
**Disease**	5.16(4.05,6.34)	3.76(3.23,4.48)	< 0.001
**Etiology**	2.27(1.97,2.61)	2.20(1.96,2.50)	0.022
**Symptoms/complications**	1.34(1.13,1.61)	1.66(1.41,1.93)	< 0.001
**Disease treatment/management**	16.96(15.61,18.85)	15.85(14.36,18.36)	< 0.001
**Total**	25.65(23.09,29.32)	23.48(21.15,27.07)	< 0.001

### Relationship between allergy rhinitis BSI and AQI in Beijing

Our study also investigated the effect of air quality on public concern about allergic rhinitis. The higher the AQI index, the worse the air quality. The trend of Beijing allergic rhinitis BSI was summarized in Figure S2 (see Additional file 5), where we found that similar to the national-wide results, at the beginning of the COVID-19 outbreak, Beijing’s public concern about allergic rhinitis was significantly lower than that in the same period of 2019. We first analyzed the correlations between each BSI of allergic rhinitis-related keywords and AQI during the one-year period before COVID-19 outbreak, and found 8 keywords with statistically significant correlations with the AQI (*p* < 0.05) (see Additional file 6, Table S3).

**Fig. 3:**
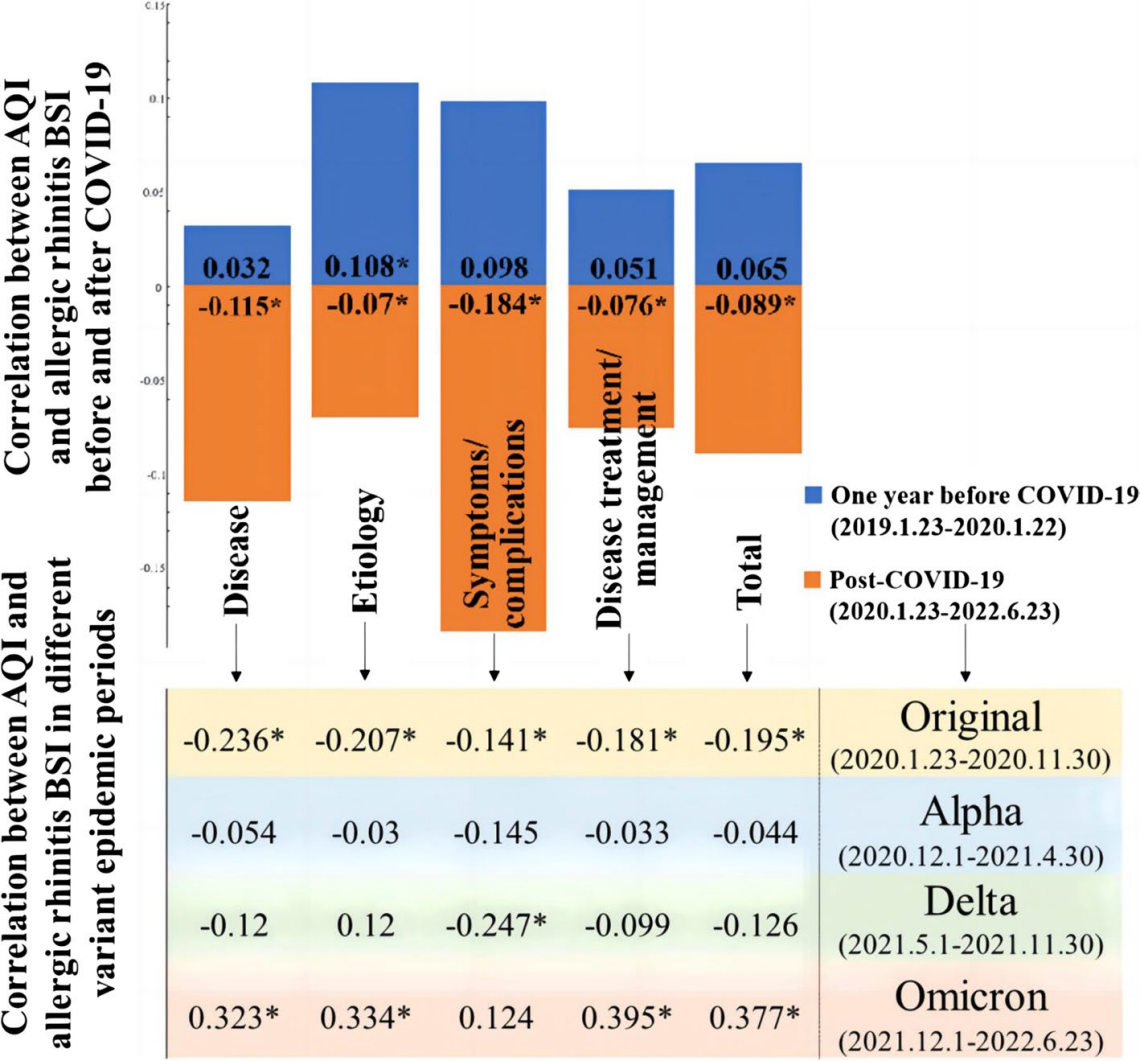
Correlations between AQI and BSI for allergic rhinitis in Beijing

Subsequently, we analyzed the association between BSIs of allergic rhinitis search behavior (both the total and the 4 themes) and AQI after COVID-19 outbreak and compared them with one year before COVID-19 outbreak. Notably, as shown in Fig. 3, there was a positive and significant correlation (*r* = 0.108, *p* < 0.05) between AQI and the BSIs of the “etiology” theme of allergic rhinitis one year before COVID-19 outbreak, but negative and significant correlations between each pair of AQI and four themes of allergic rhinitis after the occurrence of COVID-19 (*p* < 0.05). We also found that AQI was significantly negatively correlated with all allergic rhinitis themes in the Original-variant period (total: *r* = -0.195, *p* < 0.05), and was partially (not significantly) negatively associated with allergic rhinitis in the Alpha and Delta-variant periods. In the Omicron period, a higher level of AQI was associated with increased search interest of allergic rhinitis, showing a significant positive correlation (total: *r* = 0.377, *p* < 0.05). More specific descriptions can be seen in Additional file 7.

**Fig. 4:**
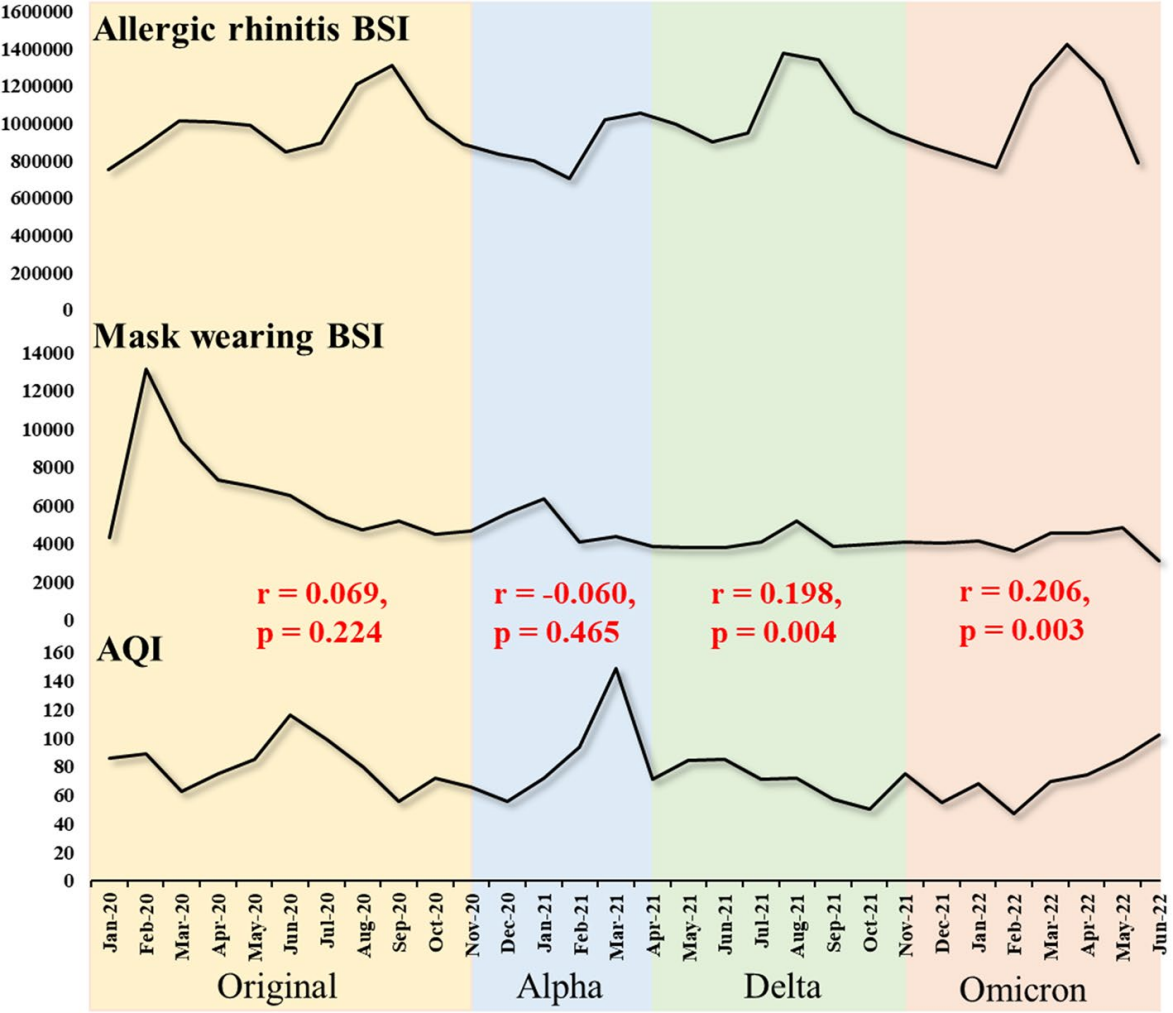
Trends of allergic rhinitis BSIs, mask wearing, and AQI in Beijing by COVID-19 variant period

We also explored the link between the search behavior of wearing masks and AQI during the four epidemic periods (Fig. 4). The result showed positive correlations between mask-wearing BSI and AQI during the Original-variant, Delta and Omicron variant dominance periods, implying that the AQI in Beijing positively affected the mask wearing BSI in these periods. However, the positive correlation coefficient was small and not significant (*r* = 0.069, *p* = 0.224) between AQI and mask wearing BSI in Original-variant period, whereas such correlation increased to a significant level in the Delta (*r* = 0.198, *p* = 0.004) and Omicron (*r* = 0.206, *p* = 0.003) variant period.

In addition, during the Omicron-variant period, the correlations between BSIs of allergic rhinitis and mask wearing showed stronger and significant positive associations (*r* = 0.585, *p* < 0.05) (see Additional file 8, Table S4). More details could be seen in Additional file 8.

## Discussion

### Principal findings

Our findings suggested that public concern about allergic rhinitis has generally declined during the COVID-19 pandemic, especially in the first half of 2020. Meanwhile, we also detected a regular seasonal component with two peaks in spring and summer for the allergic rhinitis-related internet searches in China. In addition, we examined the relationship between air quality and allergic rhinitis-related searches in the city of Beijing from 2019 to 2022. One year before COVID-19, people’s search behavior for allergic rhinitis increased as air quality became worse. However, during the COVID-19 pandemic, allergic rhinitis-related search behavior decreased with deteriorating air quality. This may be related to limited outdoor activities under the quarantine policy and widely-adopted protective behaviors such as wearing masks during the pandemic.

### Temporal trends in allergic rhinitis search behavior

Our study showed that allergic rhinitis BSI had a marked seasonal variation, which was consistent with the two previously reported peaks of airborne pollen [32]. The seasonality of allergic rhinitis search behavior may be related to both pollen and climate-changing-induced allergic rhinitis. Firstly, March to May is generally recognized as the pollen season, and pollen can act as an allergen to induce the allergic rhinitis onset [33, 34]. Secondly, from July to September, the climate is relatively hot and the frequent use of air conditioners can easily cause indoor air drying, resulting in impaired blood circulation in the nasal mucosa, which in turn triggers the onset of allergic rhinitis [35]. Therefore, along with the increased incidence of allergic rhinitis, the public's attention to allergic rhinitis also reaches its peaks.

When assessing the trend component after removing the seasonal effects, our results showed that public attention to allergic rhinitis on the Internet in China gradually increased from 2017 onwards, reaching a peak in April 2019. This was similar to previous epidemiological studies elucidated that the prevalence of allergic rhinitis maintained at a high level from 2017 to 2019 [3, 36]. Subsequently, public interest in allergic rhinitis declined in the first half of 2020, but began to rise again in the second half of that year. In summary, public concern about allergic rhinitis was significantly downward in the early stages of the outbreak, compared with before the COVID-19 outbreak in China. This might be attributed to the actions taken by the Chinese government at all levels to contain COVID-19 in the early stages of the outbreak, as preventive measures against COVID-19 virus also played an integral role in reducing the onset of allergic rhinitis caused by outdoor allergens, as confirmed in previous studies [37, 38].

### Detailed differences in public concern about allergic rhinitis before and after the COVID-19 pandemic

We carefully comparing Internet searches for the four themes of allergic rhinitis before and after the COVID-19 pandemic, the initial increase in searches of “symptoms/complications” may have been due to some overlap between early symptoms of the COVID-19 infection and respiratory symptoms in patients with allergic rhinitis [39]. Therefore, people may utilize Internet searches for rhinitis symptoms to make a preliminary differential diagnosis of the two conditions. It was also worth noting that, the search volume of the theme “symptoms/complications” had another rapid growth peak after May 2021, which was exactly when the State Council accelerated the promotion of COVID-19 vaccination nationwide. At the same time, the CDC of China released the COVID-19 Vaccination Adverse Reaction Surveillance Report. Therefore, increasing internet searches of “symptoms/complications” of allergic rhinitis may also be driven by those potential adverse reactions that people experienced or were concerned about after the COVID-19 vaccination [40], including allergic rhinitis and other allergies.

On the other hand, for “disease”, “etiology” and “disease treatment/management” themes, the implementation of the quarantine policy has restricted people’s outdoor activities and reduced their opportunities for strenuous exercise, which may subsequently reduce the incidence of allergic rhinitis triggered by excessive physical activity [41], leading to decreased search interest of allergic rhinitis and its treatment after the COVID-19 outbreak. Moreover, our results showed that pollen-related keywords “hay fever” and “pollen allergy” dropped significantly after the COVID-19 outbreak. One explanation is that in order to prevent the spread of the COVID-19 virus, people consciously maintained social distance and wore masks in public places [10, 37, 38, 42], and these measures have blocked airborne pollen, allergen exposure and cold air irritation to a certain extent, therefore effectively avoiding the onset of the disease in patients with allergic rhinitis.

### Correlation between air quality and online search behavior

In this study, the context of the COVID-19 pandemic provided us an opportunity to explore the relationship between daily air quality and public concern about allergic rhinitis, as well as their correlations with mask-wearing behavior. The correlation analysis focusing on Beijing area provided following main findings.

On the one hand, we noted that is, as air quality became worse, allergic rhinitis BSI increased accordingly before 2020, but decreased after the COVID-19 pandemic. Previous studies have shown that air pollutants can aggravate the symptoms of people with allergic rhinitis, and thus, air pollution was associated with allergic rhinitis episodes [20, 43]. Meanwhile, the implementation of national and local quarantine policies during the COVID-19 pandemic led to a reduction in human-related activities, such as industrial production and transportation, indirectly diminishing levels of urban air pollution [23, 24]. Therefore, we speculated that the effect of poor air quality on triggering allergic rhinitis may be attenuated by COVID-19 preventive and controlling measures. In the context of the COVID-19 epidemic, wearing masks can not only reduce the spread of the virus through the respiratory tract, but also simultaneously assist people with allergic rhinitis in reducing their exposure to air pollutants to cause allergic rhinitis onset [15, 44]. Therefore, the simultaneous improvements in air quality during quarantines and the adoption of mask-wearing were accompanied by a decline in search behavior related to allergic rhinitis.

On the other hand, we found that the association between air quality and allergic rhinitis BSI differed during the four different variant epidemics. Such dissimilarity was particularly pronounced during the epidemics of the Original and Omicron variants, with AQI and allergic rhinitis BSI being significantly negatively correlated during the Original-variant period but significantly positively correlation during the Omicron period. This may be explained by the changes in quarantine policies and people’s mask-wearing behavior due to the differences in transmissibility and toxicity of various variants of the COVID-19 virus [45, 46]. One possible explanation is that, at the beginning of the epidemic of the Original-variant, the mass media in China broadcasted lots of personal protection measures and urged people to wear masks, and thus most people strictly enforced the mask-wearing behavior regardless of air quality during the Original-variant period [47]. In addition, the implementation of the quarantine policy restricted also greatly increased the exposure to indoor allergic substances, leading to the incidence of allergic rhinitis [48, 49]. So even though the air quality became better in the period, people’s search behavior for allergic rhinitis increased. However, during the Omicron period, most of the mass media calls for wearing masks were “whether or not to wear a mask or not is determined based on the risk of infection” [50]. The masks were only required in public areas where crowds gathered, and the enforcement of mask wearing was not as strict as it was during the Original-variant period, while society gradually resumed production and work, and air pollution generated by human activities gradually increased. Therefore, during the Omicron period, the worse the air quality was, the more people were interested in searches of masks, and the deterioration of air quality also led to a simultaneous increase in the onset of allergic rhinitis and related internet searches.

In summary, in the context of the COVID-19 pandemic, the Baidu index reveals the potential demand of the public for allergic rhinitis-related information and mask-wearing behavior under different air quality levels. In fact, along with the liberalization of the COVID-19-related policies, personal protective behaviors will be decreased compared with the early stage of the pandemic. In this case, the health administrative department should encourage patients with allergic rhinitis not only to be aware of daily air quality, but also to wear masks accordingly to reduce exposure to allergens, especially during seasonal peak period of allergic rhinitis.

## Limitation

This study has some limitations. First, we only investigated the attention of Baidu search engine users on allergic rhinitis, without evaluating the public’s attention on other search engines or social media, which may result in bias in representing the public’s interest on the disease. Second, wearing masks can be effective in avoiding outdoor allergen exposure, but we did not evaluate the relationship between indoor allergens and allergic rhinitis search behavior due to the COVID-19 pandemic. Third, this study observed a correlation between Baidu index and AQI, however, it’s crucial to note that while correlation suggests a relationship, it doesn’t establish causation, and other confounding factors may influence this association. Future research endeavors should delve deeper into these relationships to establish a more nuanced understanding and causative links. Finally, even though a weighted filtering algorithm was employed to process the data from the Baidu index, the particular algorithm of the Baidu index has not been disclosed to the public, making it impossible to assess the index’s validity and dependability at this time. Therefore, in order to limit the impact of confounding factors on the research results and increase their objectivity, future studies should think about incorporating several search engines or social media for analysis, and the data should also be thoroughly mined.

## Conclusion

In summary, our study presented patterns of allergic rhinitis search behavior before and after the COVID-19 outbreak, where a significant declining trend was observed at the beginning of the COVID-19 outbreak. We revealed that the COVID-19 pandemic and air quality may had an impact on the online public concerns about allergic rhinitis in Beijing, as quarantines and mask-wearing behavior for the pandemic also prevented the spread of allergens and improved the air quality. Analyzing online search trends related to allergic rhinitis effectively captures users’ interests in disease information, highlighting keywords closely associated with patient symptoms and treatment needs. This valuable insight serves as a foundation for enhancing the quality of online allergic rhinitis information, offering the mass media and health departments crucial clues to refine content, so as to help Internet users acquire reliable knowledge and make informed decisions in managing allergic rhinitis. In summary, the combination of Internet users’ online search behavior can aid in monitoring allergic diseases outbreaks, reveal the needs of the population and potential patients, and facilitate designing and implementing targeted health education and promotion.

### Supplementary Information

**Additional file 1:** **Figure S1.** Allergic rhinitis keywords selection process. **Table S1.** keywords under each theme of allergic rhinitis.

**Additional file 2.**

**Additional file 3.**

**Additional file 4:** **Table S2.** The adjusted daily BSI of each of the allergic rhinitis terms before and after COVID-19.

**Additional file 5:** **Figure S2.** Allergic Rhinitis Search Trends in Beijing, from Jan 2019 to Jun 2022.

**Additional file 6: Table S3. **Associations of allergic rhinitis-related BSI and AQI during the year before covid-19 in Beijing.

**Additional file 7.**

**Additional file 8:** **Table S4.** Associations of mask wearing BSI, allergic rhinitis-related BSI and AQI in the Original-variant and Omicron variant periods in Beijing.

## Data Availability

All data are available from author Yi Yu with reasonable request.

## Abbreviations

AR: Allergic rhinitis
COVID-19: Coronavirus disease 2019
BSI: Baidu Search Index
BIS: Baidu Indexing Service
AQI: Air Quality Index
