# Supplementary material for: Online public concern about allergic rhinitis and its association with COVID-19 and air quality in China: an informative epidemiological study using Baidu index
Source: BMC Public Health. 2024 Feb 2;24:357. doi: 10.1186/s12889-024-17893-4 (PMC10837907; doi:10.1186/s12889-024-17893-4)
Supplement: Supplementary file 1 — Additional file 1: Figure S1. Allergic rhinitis keywords selection process. Table S1. keywords under each theme of allergic rhinitis. [file 12889_2024_17893_MOESM1_ESM.docx]

The identification of keywords in this study can be shown in the flowchart in Figure S1. Firstly, by using “allergic rhinitis” as the search term, we gathered weekly search terms related to allergic rhinitis in the past six months (between December 20, 2021 and June 19, 2022) from the “demand mapping” tool in the Baidu Index database. A total of 108 related terms were originally extracted. Other 17 keywords related to allergic rhinitis were included lately from literatures [1, 2]. Secondly, 27 duplicate keywords, 6 irrelevant keywords, and 4 keywords that were not recruited by the Baidu index were all removed. Finally, in order to eliminate differences in idiomatic terms, synonyms were combined and a total of 33 keywords were generated for the subsequent analysis. The 33 search terms were further grouped into the four categories of “disease", “etiology”, “symptoms/complications”, and “disease treatment/management”, which are listed in Table S1. The daily BSIs of each term were extract and used for subsequent analysis.


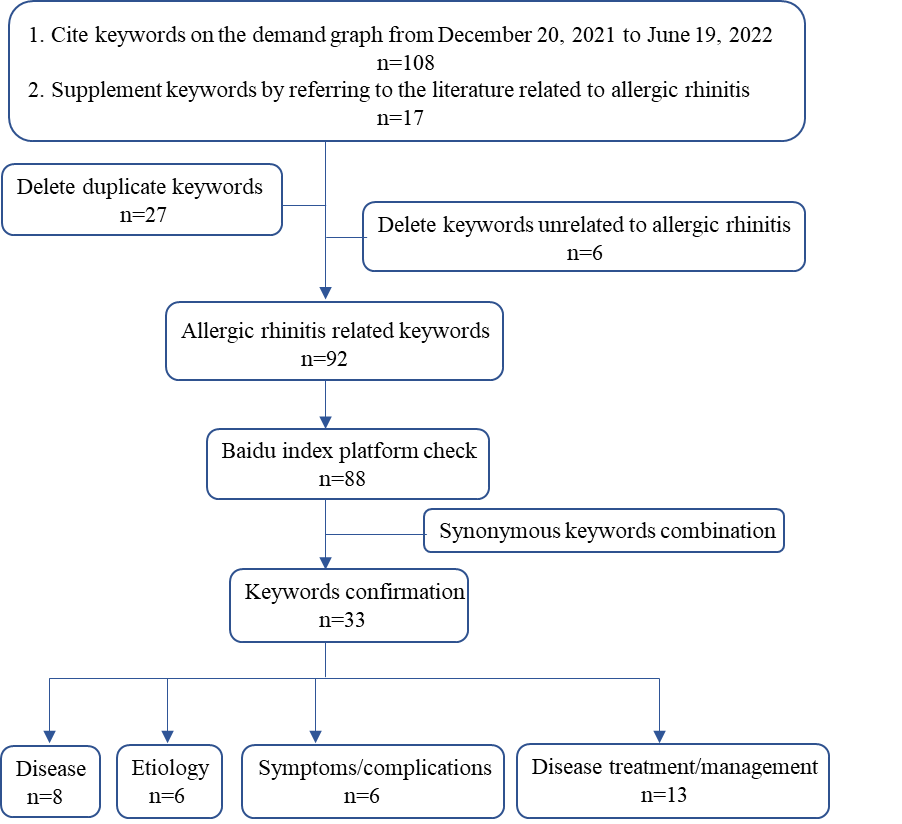


Figure S1 Allergic rhinitis keywords selection process

Table S1 keywords under each theme of allergic rhinitis

| **Themes** | **Available term in Search engine** | **English equivalent terms** |
| --- | --- | --- |
| **Disease** | 过敏性鼻炎 | Allergic rhinitis |
|  | 过敏鼻炎 |  |
|  | 什么是过敏性鼻炎 |  |
|  | 慢性过敏性鼻炎 | Chronic allergic rhinitis |
|  | 尘螨过敏性鼻炎 | Dust mites-related allergic rhinitis |
|  | 冷空气过敏性鼻炎 | Cold air-related allergic rhinitis |
|  | 季节性鼻炎 | Seasonal allergic rhinitis |
|  | 季节性过敏性鼻炎 |  |
|  | 春季 过敏性鼻炎 |  |
|  | 花粉症 | Hay fever |
|  | 小孩过敏性鼻炎 | Allergic rhinitis in children |
|  | 儿童过敏性鼻炎 |  |
|  | 幼儿过敏性鼻炎 |  |
|  | 小儿过敏性鼻炎 |  |
|  | 婴儿过敏性鼻炎 | Allergic rhinitis in infants |
| **Etiology** | 过敏性鼻炎是怎么引起的 | Causes of allergic rhinitis |
|  | 过敏性鼻炎原因 |  |
|  | 过敏性鼻炎遗传吗 | Is allergic rhinitis hereditary |
|  | 过敏性鼻炎传染吗 | Is allergic rhinitis contagious |
|  | 过敏性鼻炎会传染吗 |  |
|  | 过敏性体质 | Allergic constitution |
|  | 春天花粉过敏 | Pollen allergy |
|  | 花粉 |  |
|  | 花粉过敏 |  |
|  | 尘螨 | Dust mites allergic |
|  | 尘螨过敏 |  |
| **Symptoms/complications** | 过敏性鼻炎症状 | Symptoms of allergic rhinitis |
|  | 过敏性鼻炎 症状 |  |
|  | 过敏性鼻炎的症状 |  |
|  | 过敏性鼻炎的主要症状 |  |
|  | 过敏性鼻炎的症状有哪些表现 |  |
|  | 过敏性鼻炎咳嗽 | Allergic rhinitis cough |
|  | 过敏性鼻炎 咳嗽 |  |
|  | 经常打喷嚏 | Frequent sneezing and runny nose |
|  | 经常打喷嚏流鼻涕 |  |
|  | 过敏性鼻炎鼻塞 | Allergic rhinitis nasal congestion |
|  | 过敏性鼻炎哮喘 | Combined Allergic Rhinitis and Asthma Syndrome（CARAS） |
|  | 过敏性鼻炎 哮喘 |  |
|  | 过敏性鼻炎伴哮喘 |  |
|  | 过敏性鼻炎引发哮喘 |  |
|  | 过敏性鼻炎结膜炎 | Allergic rhinoconjunctivitls |
| **Disease treatment/management** | 过敏性鼻炎 治疗 | How to treat allergic rhinitis |
|  | 过敏性鼻炎怎么办 |  |
|  | 过敏性鼻炎怎么治 |  |
|  | 过敏性鼻炎怎么根治 |  |
|  | 过敏性鼻炎如何治疗 |  |
|  | 过敏性鼻炎如何根治 |  |
|  | 过敏性鼻炎怎样治 |  |
|  | 过敏性鼻炎解决方法 |  |
|  | 过敏性鼻炎能根治吗 |  |
|  | 过敏性鼻炎怎么治能除根 |  |
|  | 过敏性鼻炎怎么治最有效的方法 |  |
|  | 过敏性鼻炎能治好吗 | Can allergic rhinitis be cured |
|  | 过敏性鼻炎治得好吗 |  |
|  | 过敏性鼻炎好治吗 |  |
|  | 过敏性鼻炎的最佳治疗 | The best treatment for allergic rhinitis |
|  | 过敏性鼻炎的最佳治疗方法 |  |
|  | 过敏性鼻炎 中医 | Allergic rhinitis Chinese medicine treatment |
|  | 过敏性鼻炎中医治疗 |  |
|  | 过敏性鼻炎用什么药效果最好 | What medication to take for allergic rhinitis |
|  | 过敏性鼻炎吃什么药好 |  |
|  | 过敏性鼻炎用什么药 |  |
|  | 过敏性鼻炎吃什么药 |  |
|  | 过敏性鼻炎药 | Medications for allergic rhinitis |
|  | 过敏性鼻炎药物 |  |
|  | 过敏性鼻炎的药物 |  |
|  | 过敏性鼻炎 药物 |  |
|  | 过敏性鼻炎用药 |  |
|  | 治过敏性鼻炎的药 |  |
|  | 治疗过敏性鼻炎的药物 |  |
|  | 过敏性鼻炎 中药 | Allergic rhinitis herbal medicine |
|  | 过敏性鼻炎中药 |  |
|  | 抗过敏药 | Antiallergic drug |
|  | 盐酸西替利嗪片 |  |
|  | 西替利嗪 |  |
|  | 开瑞坦 |  |
|  | 雷诺考特 |  |
|  | 氯雷他定片 |  |
|  | 氯雷他定 |  |
|  | 布地奈德鼻喷雾剂 | Nasal spray |
|  | 辅舒良鼻喷雾剂 |  |
|  | 丙酸氟替卡松鼻喷雾剂 |  |
|  | 过敏性鼻炎的偏方 | Allergic rhinitis remedy |
|  | 过敏性鼻炎小偏方 |  |
|  | 过敏性鼻炎食疗 | Allergic rhinitis diet therapy |
|  | 过敏性鼻炎吃什么 |  |
|  | 过敏性鼻炎的自我疗法 | Self-treatment for allergic rhinitis |
|  | 盐水洗鼻 | Saline nasal rinses |

**References**

1. Small P, Keith PK, Kim H. Allergic rhinitis. Allergy, Asthma & Clinical Immunology 2018 14:2. 2018;14:1–11.

2. Bousquet J, Onorato GL, Oliver G, Basagana X, Annesi-Maesano I, Arnavielhe S, et al. Google Trends and pollen concentrations in allergy and airway diseases in France. Allergy. 2019;74:1910–9.
