# Supplementary material for: Online public concern about allergic rhinitis and its association with COVID-19 and air quality in China: an informative epidemiological study using Baidu index
Source: BMC Public Health. 2024 Feb 2;24:357. doi: 10.1186/s12889-024-17893-4 (PMC10837907; doi:10.1186/s12889-024-17893-4)
Supplement: Supplementary file 2 — Additional file 2. [file 12889_2024_17893_MOESM2_ESM.docx]

Beijing, as the capital city, is characterized by dense population, robust economy, and heavy traffic, cementing its position as a political and economic center of China. In recent decades, Beijing confronts a pronounced environmental exposure challenge, and is becoming one of the most stringent target areas for air pollution control measures and policies aimed at improving air quality [1, 2], making it a good surrogate to monitor variations in air quality. On the other hand, during the COVID-19 pandemic, Beijing, among the most severely impacted cities in China, demonstrated efficient implementation of measures and policies in the fight against the outbreak (lockdowns, mask-wearing mandates, vaccination campaigns, etc.). Moreover, Beijing boasts abundant educational resources, and is one of the cities with a high Internet penetration rate. With the ongoing development of network informatization, an increasing number of residents in Beijing seek information about allergic rhinitis through online platforms [3]. This unique combination of environmental challenges, effective pandemic response, and educational infrastructure makes Beijing an ideal focal point for our study.

**References**

1. Tang G, Zhao P, Wang Y, Gao W, Cheng M, Xin J, et al. Mortality and air pollution in Beijing: The long-term relationship. Atmos Environ. 2017;150:238–43.

2. Wang X, Wei W, Cheng S, Zhang C, Duan W. A monitoring-modeling approach to SO42 − and NO3− secondary conversion ratio estimation during haze periods in Beijing, China. Journal of Environmental Sciences. 2019;78:293–302.

3. LIN Feng, PANG Chong, GU Qing-long. Study on the attention of children with allergic rhinitis in Beijing based on Baidu index. Chinese Journal of Practical Pediatrics. 2021;36.
