# Supplementary material for: Online public concern about allergic rhinitis and its association with COVID-19 and air quality in China: an informative epidemiological study using Baidu index
Source: BMC Public Health. 2024 Feb 2;24:357. doi: 10.1186/s12889-024-17893-4 (PMC10837907; doi:10.1186/s12889-024-17893-4)
Supplement: Supplementary file 3 — Additional file 3. [file 12889_2024_17893_MOESM3_ESM.docx]

We further separately investigated the four themes of allergic rhinitis: “disease", “etiology", “symptoms/complications” and “disease treatment/management", and explored their seasonal fluctuations, as well as the remaining trend components after removing the seasonal variation (Fig. 2b). For all of the four themes, the seasonal components constantly showed two peaks in every March and August, the difference is that the largest search volume for “disease” and “disease treatment/management” themes occurred in August of each year while the highest BSI for the “etiology” theme occurred in March and “symptoms/complications” has two similar peaks. In terms of the trend curve, the BSI for the “disease", “etiology” and “disease treatment/management” themes showed a significant decline from January to June of 2020, while the BSI for the “symptoms/Complications” theme gradually increased and reached a peak in August 2020. After this period, the search trend for the “disease” theme gradually plateaued, and the “etiology” theme quickly climbed to its second peak in April 2021, while the BSI for the “symptoms/complications” theme gradually declined from June 2020 to March 2021 and gradually increased after May 2021, showing the opposite trend to the “etiology” theme. Meanwhile, the “disease treatment/management” theme consistently followed an identical pattern as the total BSI trend curve.
