# Supplementary material for: Online public concern about allergic rhinitis and its association with COVID-19 and air quality in China: an informative epidemiological study using Baidu index
Source: BMC Public Health. 2024 Feb 2;24:357. doi: 10.1186/s12889-024-17893-4 (PMC10837907; doi:10.1186/s12889-024-17893-4)
Supplement: Supplementary file 4 — Additional file 4: Table S2. The adjusted daily BSI of each of the allergic rhinitis terms before and after COVID-19. [file 12889_2024_17893_MOESM4_ESM.docx]

In detail, the adjusted BSI for the term “symptoms of allergic rhinitis” in “symptoms/complications” theme has the most significant increase after the occurrence of COVID-19 (p < 0.001) (Table S2). However, following the COVID-19 pandemic, public search interests in the keywords “allergic rhinitis” (in “disease” theme), as well as searches of child-specific terms, such as “allergic rhinitis in children” and “allergic rhinitis in infants", were among the most significantly decreased terms (p < 0.001). Notably, the adjusted BSI for “hay fever” (in “disease” theme; p = 0.001) and “pollen allergy” (in “etiology” theme; p < 0.001) also decreased significantly after COVID-19 outbreak (Table S2), implying that public concern or incidence of pollen-induced allergic rhinitis decreased after COVID-19. This might be associated with reduced pollen exposure and outdoor activities due to the implementation of lockdown policies and the usage of face masks.

Table S2 The adjusted daily BSI of each of the allergic rhinitis terms before and after COVID-19

| **Themes** | **English equivalent terms** | **Pre-COVID-19 (2017.01.23-2020.01.22)**  **M** | **Post-COVID-19 (2020.01.23-2022.06.23)**  **M** | **Rate of change** | **p value** |
| --- | --- | --- | --- | --- | --- |
| **Disease** | Allergic rhinitis | 3.86 | 2.45 | -36.6% | <0.001 |
|  | Chronic allergic rhinitis | 0.05 | 0.05 | 2.6% | 0.324 |
|  | Dust mites-related allergic rhinitis | 0.05 | 0.06 | 11.9% | <0.001 |
|  | Cold air-related allergic rhinitis | 0.10 | 0.27 | 158.8% | <0.001 |
|  | Seasonal allergic rhinitis | 0.33 | 0.33 | -0.4% | 0.786 |
|  | Hay fever | 0.15 | 0.14 | -3.2% | 0.001 |
|  | Allergic rhinitis in children | 0.51 | 0.39 | -24.4% | <0.001 |
|  | Allergic rhinitis in infants | 0.05 | 0.05 | -6.7% | <0.001 |
| **Etiology** | Causes of allergic rhinitis | 0.04 | 0.28 | 516.5% | <0.001 |
|  | Is allergic rhinitis hereditary | 0.05 | 0.05 | 1.9% | 0.882 |
|  | Is allergic rhinitis contagious | 0.21 | 0.19 | -8.2% | <0.001 |
|  | Allergic constitution | 0.17 | 0.15 | -12.2% | <0.001 |
|  | Pollen allergy | 1.14 | 0.90 | -21.2% | <0.001 |
|  | Dust mites allergic | 0.59 | 0.56 | -4.9% | <0.001 |
| **Symptoms/complications** | Symptoms of allergic rhinitis | 0.83 | 1.20 | 45.5% | <0.001 |
|  | Allergic rhinitis cough | 0.13 | 0.12 | -4.5% | 0.03 |
|  | Frequent sneezing and runny nose | 0.17 | 0.17 | 0.4% | 0.968 |
|  | Allergic rhinitis nasal congestion | 0.05 | 0.05 | -5.1% | <0.001 |
|  | Combined Allergic Rhinitis and Asthma Syndrome（CARAS） | 0.14 | 0.09 | -31.6% | <0.001 |
|  | Allergic rhinoconjunctivitls | 0.00 | 0.04 | - | <0.001 |
| **Disease treatment/management** | How to treat allergic rhinitis | 2.41 | 2.67 | 11.0% | <0.001 |
|  | Can allergic rhinitis be cured | 0.13 | 0.13 | 0.6% | 0.864 |
|  | The best treatment for allergic rhinitis | 0.23 | 0.28 | 26.0% | <0.001 |
|  | Allergic rhinitis Chinese medicine treatment | 0.05 | 0.05 | -8.2% | <0.001 |
|  | What medication to take for allergic rhinitis | 1.01 | 1.24 | 22.8% | <0.001 |
|  | Medications for allergic rhinitis | 0.32 | 0.35 | 9.8% | <0.001 |
|  | Allergic rhinitis herbal medicine | 0.05 | 0.07 | 42.2% | <0.001 |
|  | Antiallergic drug | 9.63 | 8.74 | -9.2% | <0.001 |
|  | Nasal spray | 1.79 | 1.56 | -12.6% | <0.001 |
|  | Allergic rhinitis remedy | 0.00 | 0.00 | - | 0.026 |
|  | Allergic rhinitis diet therapy | 0.06 | 0.06 | 0.4% | 0.243 |
|  | Self-treatment for allergic rhinitis | 0.46 | 0.24 | -47.6% | <0.001 |
|  | Saline nasal rinses | 0.76 | 0.15 | -80.5% | <0.001 |
