# Supplementary figures and images for: Online public concern about allergic rhinitis and its association with COVID-19 and air quality in China: an informative epidemiological study using Baidu index
Source: BMC Public Health. 2024 Feb 2;24:357. doi: 10.1186/s12889-024-17893-4 (PMC10837907; doi:10.1186/s12889-024-17893-4)

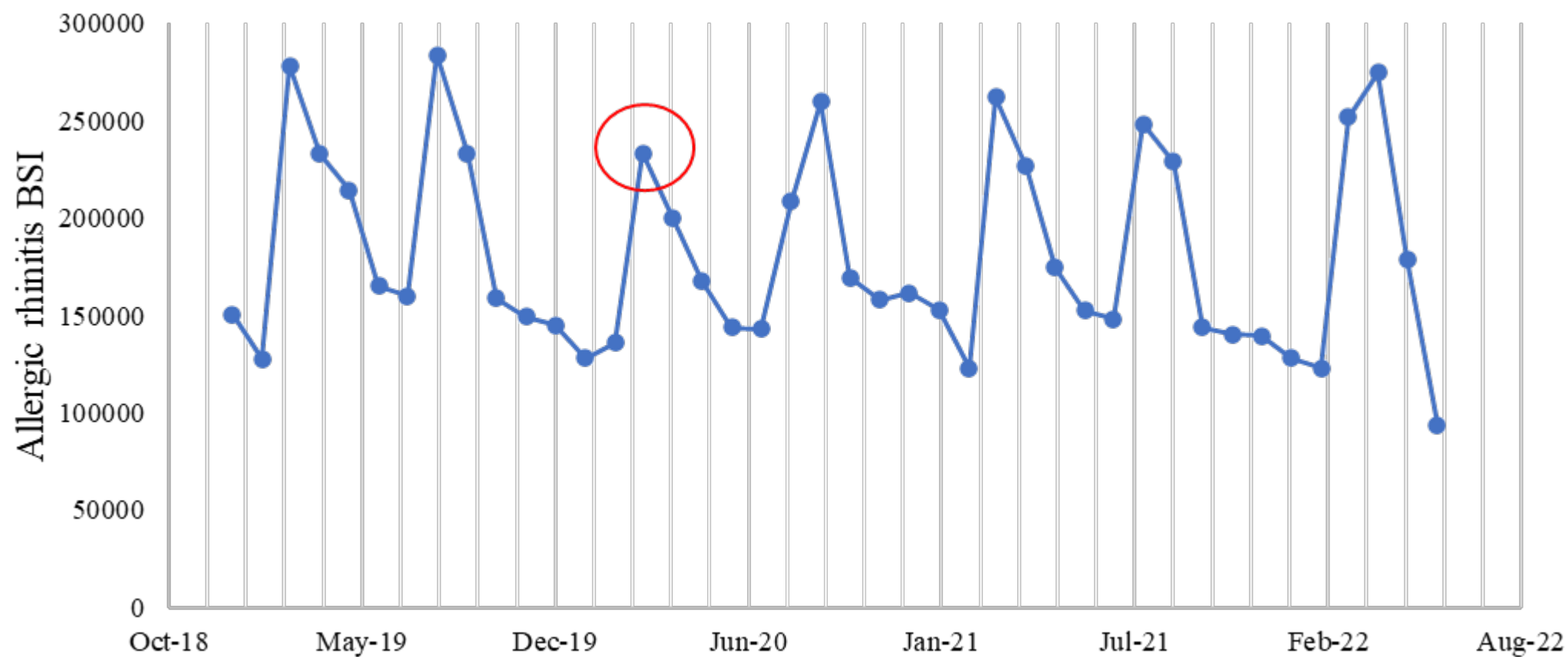

Figure S2 Allergic Rhinitis Search Trends in Beijing, from Jan 2019 to Jun 2022

Supplement: Supplementary file 5 — Additional file 5: Figure S2. Allergic Rhinitis Search Trends in Beijing, from Jan 2019 to Jun 2022. [file 12889_2024_17893_MOESM5_ESM.pdf]
