# Supplementary material for: Online public concern about allergic rhinitis and its association with COVID-19 and air quality in China: an informative epidemiological study using Baidu index
Source: BMC Public Health. 2024 Feb 2;24:357. doi: 10.1186/s12889-024-17893-4 (PMC10837907; doi:10.1186/s12889-024-17893-4)
Supplement: Supplementary file 6 — Additional file 6: Table S3. Associations of allergic rhinitis-related BSI and AQI during the year before covid-19 in Beijing. [file 12889_2024_17893_MOESM6_ESM.pdf]

Table S3 Associations of allergic rhinitis-related BSI and AQI during the year before covid-19 in Beijing

Variate	AQI
Frequent sneezing and runny nose	0.199*
Allergic rhinitis in children	0.177*
Dust mites allergic	0.174*
Cold air-related allergic rhinitis	-0.16*
Nasal spray	-0.152*
Combined Allergic Rhinitis and Asthma Syndrome（CARAS）	0.135*
Dust mites-related allergic rhinitis	0.118*
How to treat allergic rhinitis	0.107*

*p<0.05
