# Supplementary material for: Online public concern about allergic rhinitis and its association with COVID-19 and air quality in China: an informative epidemiological study using Baidu index
Source: BMC Public Health. 2024 Feb 2;24:357. doi: 10.1186/s12889-024-17893-4 (PMC10837907; doi:10.1186/s12889-024-17893-4)
Supplement: Supplementary file 7 — Additional file 7. [file 12889_2024_17893_MOESM7_ESM.docx]

To further explore the association between allergic rhinitis search index and AQI, we also conducted the correlation analysis separately under four variant epidemic periods during the COVID-19 pandemic. The results (Fig. 3) showed that AQI was significantly negatively correlated with all allergic rhinitis themes in the Original-variant period (total: r = -0.195, p < 0.05), and was partially (not significantly) negatively associated with allergic rhinitis in the Alpha and Delta-variant periods. In the Omicron period, a higher level of AQI was associated with increased search interest of allergic rhinitis, showing a significant positive correlation (total: r = 0.377, p < 0.05). That is, as air quality deteriorated, Beijing citizens had more concerns about allergic rhinitis in the Omicron period. This indicated that, in both the Original-variant and Omicron-variant periods, the AQI had a significant effect on the daily BSI of allergic rhinitis in Beijing (p < 0.05), but in opposite directions. Furthermore, during the Original-variant period, the coefficient of negative correlation attained its largest absolute value in the “disease” theme of allergic rhinitis (r = -0.236, p < 0.05), while during the Omicron-variant period, the positive correlation reached its maximum at the “disease treatment/management” theme (r = 0.395, p < 0.05).
