# Supplementary material for: Online public concern about allergic rhinitis and its association with COVID-19 and air quality in China: an informative epidemiological study using Baidu index
Source: BMC Public Health. 2024 Feb 2;24:357. doi: 10.1186/s12889-024-17893-4 (PMC10837907; doi:10.1186/s12889-024-17893-4)
Supplement: Supplementary file 8 — Additional file 8: Table S4. Associations of mask wearing BSI, allergic rhinitis-related BSI and AQI in the Original-variant and Omicron variant periods in Beijing. [file 12889_2024_17893_MOESM8_ESM.docx]

In addition, the Spearman correlations between BSIs of allergic rhinitis and mask wearing were calculated (Table S4). Similar to the results between the AQI and mask-wearing BSI, during the Original-variant period, the search behavior of mask wearing was positively correlated with allergic rhinitis BSI with no significance (r < 0.1, p > 0.05), while during the Omicron-variant period, public interests of wearing masks showed stronger and significant positive associations with both the total BSI (r = 0.585, p < 0.05) and four themes of allergic rhinitis.

Table S4 Associations of mask wearing BSI, allergic rhinitis-related BSI and AQI in the Original-variant and Omicron variant periods in Beijing

| Variate | Original  (2020.1.23-2020.11.30) | | Omicron  (2021.12.1-2022.6.23) | |
| --- | --- | --- | --- | --- |
|  | AQI | Mask wearing | AQI | Mask wearing |
| Disease | -0.280* | 0.086 | 0.323* | 0.515* |
| Etiology | -0.238* | 0.053 | 0.334* | 0.560* |
| Symptoms/complications | -0.186* | 0.134* | 0.124 | 0.456* |
| Disease treatment/management | -0.214 | 0.054 | 0.395* | 0.595* |
| Total | -0.239* | 0.075 | 0.377* | 0.585* |
| Mask wearing | 0.069 | - | 0.206* | - |

*p<0.05
